# CARDIA sleep ancillary study: study design and methods

**DOI:** 10.1093/sleepadvances/zpae071

**Published:** 2024-09-26

**Authors:** Kristen L Knutson, Kathryn J Reid, Sunaina Karanth, Nathan Kim, Sabra M Abbott, Shaina J Alexandria, Katharine Harrington, S Justin Thomas, Cora E Lewis, Pamela J Schreiner, Mercedes R Carnethon

**Affiliations:** Department of Neurology, Northwestern University Feinberg School of Medicine, Chicago, IL, USA; Department of Preventive Medicine, Northwestern Feinberg School of Medicine, Chicago, IL, USA; Department of Neurology, Northwestern University Feinberg School of Medicine, Chicago, IL, USA; Department of Neurology, Northwestern University Feinberg School of Medicine, Chicago, IL, USA; Department of Neurology, Northwestern University Feinberg School of Medicine, Chicago, IL, USA; Department of Neurology, Northwestern University Feinberg School of Medicine, Chicago, IL, USA; Department of Preventive Medicine, Northwestern Feinberg School of Medicine, Chicago, IL, USA; Department of Preventive Medicine, Northwestern Feinberg School of Medicine, Chicago, IL, USA; Department of Psychiatry and Behavioral Neurobiology, University of Alabama at Birmingham, Birmingham, AL, USA; Department of Epidemiology, University of Alabama at Birmingham, Birmingham, AL, USA; Division of Epidemiology and Community Health School of Public Health, University of Minnesota, Minneapolis, MN, USA; Department of Preventive Medicine, Northwestern Feinberg School of Medicine, Chicago, IL, USA

**Keywords:** epidemiologic studies, design, epidemiologic research, sleep habits, social disparity in health

## Abstract

Sleep and circadian disturbances are common and are experienced more often by Black compared to White individuals. We conducted an observational study of sleep that was ancillary to an ongoing cohort study, Coronary Artery Disease in Young Adults (CARDIA). The goal of the ancillary study will be to examine potential determinants of sleep/circadian disparities between Black and White adults in future analyses. Herein we describe the study design and methodology. Our ancillary study coincided with the Year 35 examination of the CARDIA study and was conducted in two phases (due to the SARS-COV-2 pandemic). Phase 1 involved only questionnaires to assess chronotype, restless legs syndrome, and the household sleep environment. Phase 2 involved three additional questionnaires to assess sleep quality, daytime sleepiness and insomnia symptoms, as well as two sleep devices. Participants wore a wrist activity monitor to assess sleep–wake patterns and light levels for 7 days and a home sleep apnea test for 1 night. A subset also had devices objectively record light, temperature, and sound levels in their bedrooms for 7 days. Sample sizes ranged based on assessment from 2200 to 2400, completing Phase 1 questionnaires, 899 with valid wrist actigraphy data, and 619 with a valid sleep apnea test. The data will be part of the full CARDIA dataset, which is available to researchers.

**Statement of Significance**

Sleep health plays a critical role in overall health; however, sleep health is not equally experienced among racial and socioeconomic groups. Herein, we describe the design of an ancillary study to a large, ongoing cohort study, the Coronary Artery Disease in Young Adults study, in which we added several measures of sleep, including questionnaires, wrist actigraphy, and a home sleep apnea test. The primary aim of this ancillary study is to examine racial and socioeconomic disparities in sleep health and the associations between sleep and other health measures. The goal of this paper is to provide methodological detail for those wishing to use these data or to replicate this study design.

## Background

Sleep disturbances affect a large proportion of the US population [1, 2]. These disturbances include inadequate sleep duration, poor sleep quality, sleep disorders (e.g. obstructive sleep apnea), suboptimal timing of sleep, and high day-to-day variability in sleep duration and timing. For example, short sleep duration is one of many sleep disturbances that contribute to poor health, which is why it was added to the American Heart Association’s Life’s Essential Eight to assess cardiovascular health [3]. While short sleep receives considerable attention, other disturbances, including excessively long sleep, poor sleep quality, irregular sleep, insomnia, sleep-disordered breathing, and restless legs syndrome (RLS), negatively influence quality of life and health. Hence, healthy sleep includes multiple dimensions important for overall health and well-being [4].

The prevalence of healthy sleep differs among racial groups. Substantial prior research [5–13] describes a higher burden of sleep disturbances among non-White adults (e.g. Black, Asian, Pacific Islander), Hispanics/Latinos, and adults with fewer socioeconomic resources than their counterparts (e.g. non-Hispanic White adults and higher socioeconomic position [SEP]). A breadth of clinical, social, behavioral, and environmental factors is likely implicated in these sleep disparities.

In addition to sleep disparities, racial/ethnic minorities and lower socioeconomic groups have the highest rates of cardiovascular disease (CVD) risk factors and some CVDs (e.g. heart failure) [14]. Findings from epidemiologic studies suggest that sleep disparities account for a proportion of the racial disparities in the onset and outcomes of CVD [15, 16]. However, our ability to develop effective strategies to ameliorate disparities in sleep health hinges on identifying a comprehensive set of modifiable factors that contribute to disparities. There are gaps in our understanding of the longitudinal determinants of sleep disparities and whether these disparities contribute to disparities in CVDs.

## Hypotheses and Objectives

The objective of this ancillary study will be to identify a set of modifiable factors associated with disparities in sleep and circadian rhythms between Black and White adults, which will be tested in future analyses and manuscripts. Our overall hypothesis is that Black participants will have a greater burden of adverse health behaviors, adiposity, psychological and social stressors, and environmental insults at the neighborhood and household level that result in a higher burden of sleep and circadian disturbances as compared with White adults. In turn, these sleep and circadian disparities will interfere with hypertension management—a highly prevalent CVD risk factor among Black adults that is hypothesized to underlie racial disparities in heart failure, chronic kidney disease, and stroke.

The specific aims of the ancillary study are as follows:

Aim 1. Determine the contribution of behavioral, biological, and psychosocial characteristics over 35 years on racial disparities in sleep disturbances in middle age.

*Hypothesis 1.1*. The higher cumulative burden and earlier onset of adverse health behaviors, adiposity, psychological distress, and adverse socioeconomic conditions among Black versus White adults are associated with more sleep and circadian disturbances.

Aim 2. Identify environmental factors associated with racial disparities in intraindividual variability (day-to-day changes) in dimensions of sleep health.

*Hypothesis 2.1.* Black participants live in neighborhoods with more noisy features (i.e. roadways, railways, airports, and commercial businesses) than White participants. These features are associated with greater intraindividual variability in sleep and circadian rhythms.

*Hypothesis 2.2.* The household sleep environment for Black participants will have more bedroom noise and light pollution and a less optimal temperature, leading to greater intraindividual variability in sleep health.

Aim 3. Quantify the contribution of sleep disturbances on racial disparities in blood pressure levels.

*Hypothesis 3.1.* Black and White participants with sleep and circadian disturbances will have higher blood pressure levels and worse hypertension control.

*Hypothesis 3.2*. Sleep and circadian disturbances will partially mediate racial disparities in blood pressure control.

We will additionally explore whether gender modifies the hypothesized associations across all Aims.

## Methods

### The CARDIA study

This sleep study is ancillary to an ongoing cohort study, the Coronary Artery Disease in Young Adults (CARDIA) study, which has been described in detail previously [17]. Briefly, the CARDIA study began in 1985 and recruited young adults aged 18–30 years. Participants were balanced across race, gender, age, and education. There are four study sites: Birmingham, Alabama; Chicago, Illinois; Minneapolis, Minnesota; and Oakland, California. The current sleep ancillary study involved all four sites, and all CARDIA participants were eligible for the sleep ancillary study.

### Study design

Due to the COVID-19 pandemic, the study was conducted in two phases. The first phase began in October 2020 and included the administration of three questionnaires that participants completed remotely (i.e. by phone, US mail, or a secure internet site). The second phase occurred between May 2021 and March 31, 2023, and consisted of additional questionnaires, wrist actigraphy, and a type 3 home sleep test, as described below. At two sites, participants also received devices to collect room temperature, sound pressure, and light exposure in the primary sleeping room. Devices were distributed during the Year 35 clinical exam visit to participants who attended the exam. Alternatively, devices were mailed to participants if they did not attend the clinic exam. Table 1 details the measures and sample sizes by study phase. The University of Alabama Birmingham, institutional review board approved the study protocol, and informed consent was obtained from participants prior to data collection. Participants were compensated $10 for completing the Phase 1 questionnaires, $15 for completing Phase 2 questionnaires, $50 for wearing the two sleep devices (wrist actigraphy and the home sleep test), and $25 for using the environmental devices.

**Table 1.** Measurements and sample sizes by phase of study

Phase of study	Measurements	Sample size^*^
Phase 1	Morningness–Eveningness Questionnaire (MEQ)	2200
	Cambridge–Hopkins Restless Legs Questionnaire	2402
	Household sleep environment	2407
	Participated in at least one of Phase 1 sleep assessments	2425
Phase 2	Wrist actigraphy	899
	Home Sleep Test	619
	Pittsburgh Sleep Quality Index	1159
	Epworth Sleepiness Scale	1212
	Insomnia Severity Index	1212
	HOBO (light, temperature)	379
	Extech (sound)	354
	Participated in at least one of Phase 2 sleep assessments	1317

^*^Number with valid data (see methods for the definition of valid by measurement type).

### Data collection, processing, and quality assurance

We conducted both objective and subjective assessments of sleep health, which we describe in detail below. Staff from each site attended a training session to review procedures for initiation and maintenance of devices, participant instruction (scripts provided), and downloading and transmission of the data to the Sleep Reading Center (SRC) at Northwestern University. This training session was followed up with both a written and practical test. The practical test involved initiating and downloading each device (wrist actigraphy, home sleep test, sound recording, and light recording device), and then transferring the files electronically to the SRC. The SRC then reviewed the written and practical test files and certified the staff if these procedures were completed correctly. Importantly, only unique study identification numbers were used for participants when initializing devices, labeling paper forms and entering data. Thus, the only protected health information included in shared data are the dates of participation.

### Objective sleep assessments

#### Wrist actigraphy.

Participants were instructed to wear a wrist actigraphy device (Actiwatch Spectrum PLUS by Philips Respironics, Murrysville, PA) on the nondominant wrist for 7 days and complete a daily sleep log provided to the participant on paper. The actigraphy device collected both light (red, green, blue [RGB]) and activity data. Participants were instructed to wear the device continuously, day and night (including while washing or taking a shower). Participants were instructed to press a button, the “event marker,” to indicate when they were going to sleep and when waking up. In parallel, participants were asked to complete a daily sleep log. This daily sleep log required participants to indicate the date, bedtime *last night*, wake time *today*, whether it was a free day or not (no work or school), and whether they napped. Sleep log data were entered into the electronic data capture system by staff at each site.

Staff at each site sent a copy of the sleep log and the actigraphy data to the SRC at Northwestern University for processing via secure file transfer. Actigraphy data were processed using Actiware Software (version 6.2.0.39). Prior to the placement of rest intervals (scoring), the SRC personnel checked several key metrics to ensure devices were initiated correctly, including epoch length (30 seconds), recording setting “(RGB) and activity,” and time zone. The study was also reviewed for quality including wear time and device failure.

**Figure 1.**
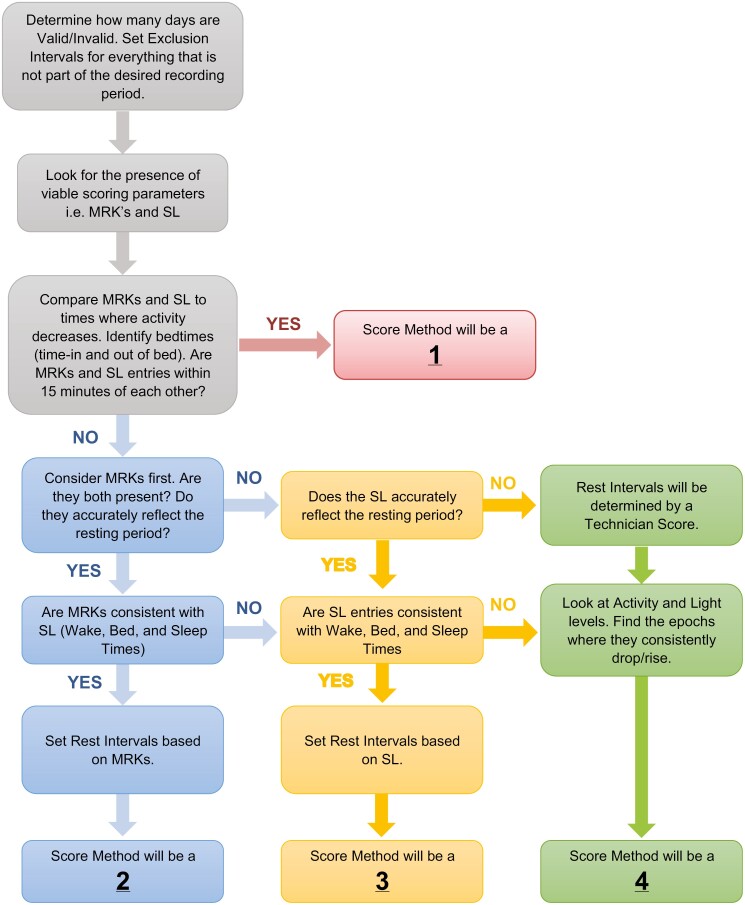
Flow chart describing how rest intervals were scored in the wrist actigraphy data. MRK = Event Marker; SL = Sleep Log.

To score the data, the SRC followed a series of predetermined rules to set the start and end of a rest interval based on the presence and hierarchical order of specific parameters (see Figure 1) [18]. The order in which parameters were considered was as follows: (1) event markers; (2) sleep log; and (3) activity/light information. The method used to assign rest start or end was coded using the following rubric: (1) event marker and sleep log (both present and within 0–15 minutes of each other); (2) event Marker; (3) sleep log; and (4) technician (when no event marker or sleep log available, in which case activity/light level used). All intervals with technician scoring were reviewed by SRC staff and approved by the SRC director. Setting rest intervals for naps followed similar guidelines; naps were only scored if indicated by an event marker and/or sleep log. Naps that were within 30 minutes of the primary rest interval were considered part of the primary rest interval and scored as such. All scored records were reviewed by the SRC director weekly and then monthly with the principal investigator for final approval.

Key sleep characteristics for each participant with a minimum of four primary rest intervals were averaged and included time in bed, total sleep time, sleep start time, sleep end time, sleep percentage, fragmentation index, wake after sleep onset, sleep midpoint, and sleep regularity index [19]. Key rest-activity metrics for the 24-hour activity data included the number of valid 24-hour periods, interdaily stability, and interdaily variability [20, 21].

#### Home sleep test.

Participants wore the Nox T3s device (Nox Medical, Reykjavik, Iceland), which is an at-home polysomnography test, for 1 night. Participants were instructed to use this device on the first night that they wore their Actiwatch. The device includes one nasal cannula (airflow), two respiratory inductance plethysmography (RIP) belts (respiratory effort), and a pulse oximeter. The associated software (Noxturnal) is used to configure the device and review/process data. Staff at each site initialized the device for each participant and explained how to wear the device. Furthermore, a video tutorial was made available to participants for viewing at home.

Upon return of the device, the Nox data were downloaded by staff at each site and then transferred to the SRC for review and processing. Noxturnal software automatically analyzed the sleep recording after download, after which a sleep technician reviewed the recording. Specifically, the technician would confirm the start and end time of the analysis window, which would correspond to bedtime and waketime. After setting the analysis window, the sleep technician checked the signal quality for the four signals: oxygen saturation (SpO_2_), airflow, abdomen respiratory inductance plethysmography (RIP), and thorax RIP. If the SpO_2_ signal quality was below 85%, the study was considered invalid. If the airflow signal quality was 80%–85%, it was reviewed by the study sleep physician, who determined the validity of the study and analysis settings. If the airflow signal quality was below 80%, the technician used the alternative respiratory scoring method described below. We used two analysis protocols in Noxturnal to automatically score respiratory events depending on signal quality. The primary method used all signals (airflow, RIP, and oximetry) to identify apneas and hypopneas. The alternative method was used when airflow signal quality was poor and used only RIP and oximetry. Apneas and hypopneas were not manually scored. Apneas were defined as >90% drop in airflow that lasted at least 10 seconds. Hypopneas were defined as a ≥30% decrease in airflow for 10 seconds associated with a ≥4% reduction in oxygen saturation or arousal.

The primary measures obtained from the Nox device are apnea–hypopnea index and oxygen desaturation index. Other variables exported from this device include counts of apneas and hypopneas, both total and by body position (supine vs. nonsupine), as well as duration of time that blood oxygen was below specific thresholds (i.e. 90%, 88%, and 85%).

### Subjective sleep assessments

Participants completed several questionnaires: some were completed during Phase 1 and some during Phase 2 (see Table 1).

#### Morningness–Eveningness Questionnaire (MEQ).

The MEQ captures circadian preference, that is, when people prefer to do certain activities [22]. It has 19 items that ask the respondents about the time of day they would prefer to wake up and go to bed or to exercise or work, as well as how they feel after waking or in the evening. Scores range from 16 to 86, and lower scores indicate greater eveningness (i.e. prefer evenings). Scores can also be grouped into the following categories: definitely morning types (70–86 points), moderately morning types (59–69 points), neither type (42–58 points), moderately evening type (31–41 points), and definitely evening types (16–30 points).

#### Cambridge–Hopkins Restless Legs Questionnaire (CHQ-RLS).

This instrument has 13 items and screens for RLS [23]. The CHQ-RLS questionnaire has a sensitivity of 87.2% and a specificity of 94.4% for determining RLS [23].

#### Household sleep environment.

We developed a 30-item questionnaire to identify characteristics of the sleep environment and behaviors that may impact sleep health. The questions include characteristics of the home (e.g. detached home, apartment, etc.), household size, location of the sleeping room within the home, presence of air-conditioning, age of mattress, typical sleep position, presence of pets, presence of children, noise, light, sleep-related behaviors (e.g. caffeine use or exercising within 2 hours of bed; activities in bed, such as reading, watching TV, or eating), presence of bed partner and their behaviors (e.g. going to bed at similar time as respondent).

#### Pittsburgh Sleep Quality Index (PSQI).

The PSQI is a 19-item instrument designed to assess subjective sleep quality over the past month [24, 25]. It includes 19 questions from which seven component scores (subjective sleep quality, sleep latency, sleep duration, habitual sleep efficiency, sleep disturbances, use of a sleep medication, and daytime dysfunction) are calculated and summed into a global score [24]. The global score ranges from 0 to 21, and higher scores indicate worse sleep quality. In the original validation paper, the component scores and individual items demonstrated high internal consistency (Cronbach’s alpha = 0.83 for both) [24].

#### Epworth Sleepiness Scale (ESS).

[26, 27] The ESS assesses the level of daytime sleepiness based on eight questions that ask the respondent the likelihood that he/she/they would fall asleep in specific situations on a four-point scale ranging from “no chance” to “high chance.” The scores range from 0 to 24, and higher scores indicate greater sleepiness [26].

#### Insomnia Severity Index (ISI).

[28] The ISI is a seven-item questionnaire to screen for insomnia and assess the severity of insomnia symptoms [28]. The responses to all questions are on a five-point Likert scale, and responses are summed for a final score that corresponds to four categories: no clinically significant insomnia, subthreshold insomnia, clinical insomnia (moderate), and clinical insomnia (severe).

### Blood pressure measures

Resting systolic and diastolic blood pressure was collected from participants who had been resting in the seated position for at least 5 minutes using the Omron HEM907XL automated blood pressure monitor. Participants’ arm circumference was measured to determine the correct cuff size. Blood pressure measurements were collected in triplicate, with a 30-second rest period between each measurement; the final two measurements were averaged. Normal, elevated, Stage 1 and Stage 2 hypertension were determined based on the following criteria designated in the 2017 American College of Cardiology/American Heart Association (ACC/AHA) Blood Pressure Guidelines: <120/<80 mmHg, 120–129/<80 mmHg, 130–139 or 80–89 mmHg, ≥140 or ≥90 mmHg, respectively [29]. For our analyses, participants who reported using medications were determined as having hypertension, and the designation of Stage 1 or Stage 2 was based on measured blood pressure even if taking medication. Blood pressure control was dichotomized as a blood pressure target <130/80 mmHg according to ACC/AHA [29] and categorized over three levels according to 2023 European Society of Hypertension guidelines [30] <130/80 mmHg, 131–140/81–90 mmHg, and >140/90 mmHg.

### Environmental measures

In two of the CARDIA sites, Birmingham, AL, and Chicago, IL, we asked participants to put two devices in their primary sleeping room for the 7-day period during which they were wearing the wrist activity monitor. These devices included the HOBO (light, temperature) and the Extech (sound pressure levels), which are described below. Participants were instructed to place these devices near the bedside, but not directly under a lamp.

In addition to the sleep environment, there is evidence that light exposure across the 24-hour day may impact sleep and health. The wrist actigraphy device (described above) also has light sensors that measure red, green, and blue light levels (µW/cm^2^) throughout the recording. These light measures are then combined within the Actiware Software (version 6.2.0.39) to estimate “white light” levels (lux). This estimate of light is available from all CARDIA sites.

#### HOBO light and temperature monitor.

The HOBO Pendant MX Temp/Light logger (Onset Brands, Bourne, MA) is a compact, waterproof device that measures temperature and light in indoor and outdoor environments. The HOBO device measures temperature from around −20 to + 70°C and light levels in lux from 0 to 167 731 lux. The device stored temperature and light data throughout the 7 days, which was downloaded by study staff after the device was returned. The settings used in this study included a logging interval of one minute. Start date logging settings were configured to start logging data at 8 am Central Time once the devices were received, and the devices were set to stop logging 10 days from the start date to get 7 full days for sleep assessment and analysis. Primary variables include mean light and temperature levels during the sleep period, mean 24-hour light levels, and time above 0 lux or 10 lux [31–33].

#### Extech Sound Meter.

The Extech Sound Level Meter USB Datalogger Model 407760 (Teledyne FLIR, Wilsonville, OR) is a sound meter that records and stores sound pressure levels in decibels (dB) in the nearby environment. The Extech meter can measure and store 129 920 readings and comes with Windows-compatible software, allowing for configuration and data download. The Extech device can measure sounds between 30 and 130 dB with an accuracy of ±1.4 dB. The following parameters were used to initialize the Extech: 1-minute sampling rate, 30 240 sampling points, a low level of 0 and a high level of 130 for the alarm sound, and the selection of the STOR, dBA, SLOW, and manual settings. For analyses, we will focus only on the sleep period intervals based on actigraphy since that is when the environmental factors could influence sleep. The primary variables extracted from these devices are mean sound levels during the sleep period and the time above 60 dB, which is approximately the level of sound for normal conversation.

### Statistical analysis

The general analytic plan for our future analyses will be similar for all study aims. In each aim, we will investigate which characteristics may explain racial disparities in the outcome. We will first identify which outcomes vary by race using regression and machine learning techniques. Then, we will compare pairs of nested regression models to estimate the percentage of the racial disparity in the outcome explained by each characteristic. Each model pair will contain a base model and a full model. The base model will include race and other covariates (e.g. gender, age). The full model will add the characteristic(s) of interest to the base model. The coefficient estimate for race and the accompanying 95% confidence interval from the adjusted model will be reported. The coefficient estimates for race will also be used to calculate the percent reduction in the coefficient estimate for race. The percent reduction is interpreted as the percent of the racial disparity in the outcome “explained” by the characteristic. Given known differences in habitual sleep, sleep disturbances, and circadian rhythms by gender that are attributable to differences in biology, aging, and social roles between men and women, all analyses will test for effect modification by gender using interaction terms. Sensitivity analyses will use multiple imputations to impute missing data to account for differential missingness by race, sex, age, and education. Secondary analyses will repeat this process using socioeconomic indicators instead of race to assess whether the associations between socioeconomic indicators and outcomes are attenuated after adjusting for characteristics. Exploratory analyses will use other statistical techniques (e.g. the gap closing estimand [34], mediation analysis) to evaluate whether some characteristics explain the racial disparities in sleep and health outcomes.

For Aim 1, linear and logistic regression models will be used to evaluate the contribution of behavioral, psychosocial, and clinical characteristics on racial disparities in habitual sleep and circadian disturbances, as measured by questionnaires and 7-day averages of objective sleep data. Since CARDIA collected data on several of these characteristics at multiple previous exam years, we will use three modeling strategies to characterize changes in the risk factors over time: (1) cross-sectional analyses using only the characteristics measured at the Y35 exam; (2) analyses using the cumulative burden of each characteristic, calculated by summing measurements across exam years and multiplying by elapsed time (e.g. pack-years of smoking); (3) trajectory analyses to identify patterns in characteristics over time. For Aim 2, we will assess the extent to which objectively measured environmental factors (i.e. light, temperature, sound) explain racial disparities in objectively measured sleep outcomes. We will use linear mixed-effect models with a random effect for a night with this aim to account for within-person correlation across the seven nights of the study. In Aim 3, we will use sleep metrics as explanatory characteristics in models of blood pressure control among participants with hypertension. These models will estimate the percentage of the racial disparity in blood pressure control in participants with hypertension that can be explained by environmental characteristics.

## Results

### Data received

At the Year 35 CARDIA examination, there were 2506 participants included in the remote assessments during Phase 1 (54.7% of the surviving cohort), 2212 participants examined during the in-person clinical examination in Phase 2 (48.5% of the surviving cohort), and 3019 participants who participated in any part of the Year 35 exam. Of these 3019, 2733 participated in at least one part of the sleep ancillary study. Sample sizes for each type of sleep data by study phase are presented in Table 1. We collected three questionnaires during Phase 1 (i.e. MEQ, CHQ-RLS, Household Sleep Environment). The other questionnaires (i.e. ESS, PSQI, ISI) and the devices were administered during Phase 2. In Phase 1, 2425 participants completed at least one questionnaire. In Phase 2, we received a total of 720 Nox recordings, of which 619 (86%) were considered valid recordings. We received 961 Actiwatch files, of which 899 (93.5%) were considered valid recordings. Only two sites distributed the environmental devices, and we received 379 HOBO files and 354 Extech files (there was no definition of “valid” for these files). In Table 2, we present data counts by site since there may be regional differences that impact sleep. In Table 3, we provide the number of participants by maximum number of days of data. For example, there are 71 participants with a maximum of six valid primary sleep periods (“days”) and 412 with seven valid primary sleep periods. Table 3 also provides the number of days of overlap between the environmental measures and the valid actigraphy data. For example, 125 have a maximum of 7 days where the Hobo (temperature, light) and actigraphy overlap.

**Table 2.** Sample sizes by site for each data type

Measurements	Birmingham, AL	Chicago, IL	Minneapolis, MN	Oakland, CA
Morningness–Eveningness Questionnaire (MEQ)	551	419	603	627
Cambridge–Hopkins Restless Legs Questionnaire (RLS)	601	449	689	663
Household sleep environment	596	446	688	677
Wrist actigraphy				
Files received	193	311	274	183
Valid files	171 (89%)	286 (92%)	268 (98%)	174 (95%)
Home Sleep Test				
Files received	140	219	205	156
Valid files	125 (89%)	185 (84%)	184 (90%)	125 (80%)
Pittsburgh Sleep Quality Index (PSQ)	211	351	268	329
Epworth Sleepiness Scale (ESS)	222	354	274	362
Insomnia Severity Index (ISI)	224	353	275	360
HOBO (light, temperature)	150	229	n/a	n/a
Extech (sound)	146	208	n/a	n/a

**Table 3.** Number of participants with 1–8 days of valid actigraphy data as well as the number of days of overlap between valid actigraphy and two devices measuring environmental factors

Maximum number of days of data	Wrist actigraphy^*^	Hobo (temperature, light)^b^	Extech (sound)^†^
0	47	554	628
1	1	2	7
2	8	5	7
3	6	3	7
4	9	6	9
5	21	9	8
6	71	38	31
7	412	125	95
8	386	169	119

^*^Days are defined as the number of valid primary sleep periods.

^†^Number of periods of recording that overlap with the primary sleep period. Note only two sites (Birmingham, AL, and Chicago, IL) distributed these devices.

### Demographics

Table 4 summarizes the age, gender, race, and educational distribution in the Phase 1 and Phase 2 samples. The mean age was 61 years; approximately 60% of our sample were women and 43% were Black in our ancillary sample. On average, participants had 15 years of education. In terms of highest degree earned, 28.1% had a high school degree or GED, 13.0% had an associated degree, 26.5% had a bachelor’s degree, 23.4% had a master’s degree or higher, and 2% had less than a high school degree. Table 4 also compares the ancillary study participants to those participants who were part of the full Y35 core exam sample but did not participate in this ancillary study. Those who did not participate in either phase of the sleep ancillary study were more likely to be male or Black, and those who did not participate in Phase 2 of the ancillary study were also younger and had less education.

**Table 4.** Comparison of characteristics between participants who completed each phase of the CARDIA Sleep Study and participants who completed the Year 35 CARDIA examination

	Phase 1 (questionnaires)^†^		Phase 2 (devices and questionnaires)^†^	
	Included	Excluded^‡^	Included	Excluded^§^
*N*	2425	594	1317	1702
	Mean (SD) or *n* (%)	Mean (SD) or *n* (%)	Mean (SD) or *n* (%)	Mean (SD) or *n* (%)
Age (years)	61.1 (3.6)	61.0 (3.7)	61.4 (3.6)^*^	60.8 (3.6)^*^
Education level (years)	15.1 (2.7)	15.3 (2.6)	15.4 (2.6)^*^	14.9 (2.7)^*^
Sex (% female)	1433 (59.1)^*^	302 (50.8)^*^	806 (61.2)^*^	929 (54.6)^*^
Race (% Black)	1053 (43.4)^*^	298 (50.2)^*^	568 (43.1)	783 (46.0)

^†^See Table 1 for study components by phase.

^‡^Participants who completed either phase of the Year 35 examination but did not complete Phase 1 sleep questionnaires.

^§^Participants who completed either phase of the Year 35 examination but did not complete Phase 2 sleep devices and questionnaires.

^*^ *p* < .05.

## Discussion

The overall goal of this study was to collect multiple dimensions of sleep and circadian health using wrist actigraphy, a home sleep test, and self-reported questionnaires. Our aim is to identify determinants of sleep disparities between racial groups, including socioeconomic, behavioral, biological, psychosocial, and environmental characteristics. Furthermore, we plan to examine whether sleep health is associated with hypertension control. Of note, there are numerous assessments collected as part of the CARDIA study, including 35 years of data collection preceding or coinciding with this ancillary study, which provides opportunities for the examination of novel research questions. All assessments conducted at each wave of the CARDIA study are described on the CARDIA website (see below). Currently, associations between sleep measures and incident disease cannot be ascertained, however, it is anticipated that the CARDIA study will continue to follow participants over time, which would allow for prospective analyses in the future.

### Strengths and limitations

The strengths of this project include the objective assessment of habitual sleep patterns using wrist actigraphy as well as the objective assessment of sleep apnea using a home sleep test. In addition, these data were collected as part of an ancillary study of a large, ongoing cohort studied for 35 years. One limitation is that these objective sleep data have not been collected longitudinally at all sites, which limits our ability to investigate changes in sleep in a single site (Chicago) where sleep measures were captured via actigraphy in 2003–2004 [6]. In addition, the COVID-19 pandemic delayed the initiation of this study which reduced participation rates in the core study and ancillary study. For example, only 49.3% of the surviving cohort participated in the in-person elements of the Y35 examination, as compared to 71% of surviving participants completing the prior two (Y25 and Y30) examinations. Nonetheless, these data will provide a unique opportunity to explore associations between sleep health and sociodemographic and clinical characteristics.

## Data Availability

More details about the CARDIA Study and associated documentation are available at: https://www.cardia.dopm.uab.edu/. CARDIA also deposits data into the NHBLI’s Biologic Specimen and Data Repository Information Coordinating Center (BioLINCC) website (https://biolincc.nhlbi.nih.gov/home).

## Future Directions

In addition to addressing the specific aims of the funded study, we anticipate that these data will provide novel avenues of research. These data will be deposited into the CARDIA data repository and made available to approved researchers through the CARDIA study data sharing procedures. Interested investigators are encouraged to review protocols and data available on CARDIA’s website and review procedures for applying for access.
